# Homologous chromosome pairing starts at the ends

**DOI:** 10.1093/plphys/kiae247

**Published:** 2024-05-07

**Authors:** Joke De Jaeger-Braet

**Affiliations:** Assistant Features Editor, Plant Physiology, American Society of Plant Biologists; Department of Developmental Biology, Institute of Plant Science and Microbiology, University of Hamburg, Hamburg 22609, Germany

Flowering plants reproduce sexually, which involves the production of male and female haploid gametes from diploid pollen mother cells and megaspore mother cells, respectively. The male gametes, also called sperm cells, will subsequently fertilize the female gametes, the egg cell and central cell, which is followed by embryogenesis (Hafidh and Honys 2021). One of the essential processes for sexual reproduction is meiosis, 1 round of DNA replication followed by 2 rounds of chromosome segregation. Fundamental during meiosis is the exchange of genetic information, which occurs through meiotic recombination between homologous chromosomes (homologs). In addition, the formation of at least 1 crossover, the product of meiotic recombination, between the homologs is required for proper chromosome segregation (Zickler and Kleckner 2023).

To ensure a successful meiotic division, it is crucial that the homologs pair properly and form connections. Homologous pairing is not trivial when all homologs have to simultaneously co-align along their entire length without entangling one another. Pairing is especially challenging for species with many and/or long chromosomes. During meiotic prophase, chromosomes are organized in a linear array of DNA loops. This conserved chromosome structure is essential for pairing, meiotic recombination, synapsis (the stabilization of paired homologs), and chromosome segregation (Morgan et al. 2023). How the homologs find each other and pair involves different highly coordinated processes that are not completely conserved among species, and in several species not all mechanisms are fully understood yet (Tian et al. 2024). In this issue of *Plant Physiology*, You et al (2024) provide a comprehensive dissection of homolog pairing initiation and its requirements in rice (*Oryza sativa*), using fluorescence in situ hybridization assays.

Chromosomes undergo a complete reorganization during prophase, which facilitates pairing. In most species, the structural elements required for this reorganization are mainly the chromosome ends, also called telomeres. You et al. (2024) show the importance of the telomeres in pairing in rice. First, the chromosomes are rather randomly distributed in the nucleus (Fig. A). Subsequently, the telomeres attach at the nuclear envelop via a linker of nucleoskeleton and cytoskeleton complex (Fig. B). This complex drives the clustering of telomeres by which they polarize at one side of the nucleus to form a so-called chromosome bouquet or telomere bouquet (Fig. C) (Ding et al. 2007; Yoshida et al. 2013). This specific bouquet configuration occurs in most species and facilitates chromosome movement and homology search of the far-end chromosomal regions (Scherthan 2001; Harper et al. 2004). Mutants of genes involved in chromosome reorganization cause severe meiotic defects—for example, chromosome entanglements and tremendous reduction in fertility—showing the essential role of nuclear reorganization during meiosis (Varas et al. 2015). In rice, the telomeres aggregate at the nuclear envelope in early prophase and around the nucleolus in mid prophase (Fig. B and C) (Zhang et al. 2017; You et al. 2024). Mutants that fail to form the telomere cluster at the nuclear envelop have problems in homologous pairing, synapsis, and crossover formation (Zhang et al. 2017). Further, the telomere aggregation around the nucleolus shown by You et al. is very peculiar, and its potential role should be investigated in the future.

**Figure.**
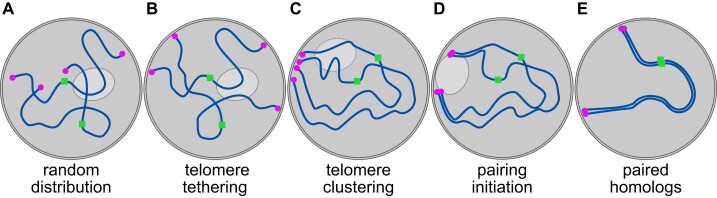
Chromosome reorganization and homologous pairing during meiotic prophase in rice. Schematic representation of the different steps to achieve homologous pairing in the nucleus of rice meiocytes (cells undergoing meiosis). **A to E)** Telomeres (magenta circles) and centromeres (green squares) are highlighted for 1 pair of homologous chromosomes (dark blue threads) and the nucleolus (in light grey).

To address the hypothesis if the chromosome reorganization, especially the bouquet formation, facilitates pairing in rice, You et al. painted the chromosome arms during meiotic prophase. They could show that once telomeres are tethered and polarized at the nuclear envelop, homologous pairing gets initiated from both chromosome ends simultaneously. The paired regions gradually extend toward the chromosome center and centromeric region (Fig. D). Thereafter, telomeres progressively separate more again and eventually, fully paired homologs are formed with telomeres and centromeres per chromosome pair separated (Fig. D and E) (Zhang et al. 2017; You et al. 2024). With these experiments, You et al. visualize very precisely that homologous pairing in fact is initiated at the chromosome ends.

You et al. also revealed the requirements of the chromosome ends for clustering and homologous pairing initiation by using different chromosome variants with modified ends. The native ends and distal regions are required for the recognition of the homologs and the initiation of pairing. Neotelomeres, which are telomeres added to heal broken chromosomes, have the capacity to form the bouquet configuration but lack the capacity to initiate pairing. In addition, if one end is lost, pairing can still be completed by initiation from the remaining native end (You et al. 2024). You et al. uncovered the plasticity of telomere clustering and chromosome pairing in rice, which is most likely also the case in most organisms.

In contrast to some other species, the centromere localization in rice remains random during chromosome reorganization until late prophase (Fig. B and C) (Zhang et al. 2017; You et al. 2024). In fission yeast (*Schizosaccharomyces pombe*) and maize (*Zea mays*), for example, the centromeres associate at the opposite side of the telomeres during early prophase. In that case, those centromeric interactions are also involved in pairing (Da Ines and White 2015).

The work of You et al. provides a clear picture of homologous pairing initiation and its requirements during rice meiosis, which stimulates the further discovery of the underlying molecular mechanisms—for example, how homology search is accomplished at the telomere cluster.

## Data Availability

There are no new data associated with this article.
